# Targeting Recycling Endosomes to Potentiate mRNA Lipid Nanoparticles

**DOI:** 10.1021/acs.nanolett.3c04415

**Published:** 2024-04-19

**Authors:** Jeehae Shin, Cameron J. Douglas, Shanwen Zhang, Ciaran P. Seath, Huan Bao

**Affiliations:** † Department of Molecular Physiology and Biological Physics, University of Virginia, 480 Ray C. Hunt Drive, Charlottesville, 22903 Virginia, United States; ‡ Department of Molecular Medicine, UF Scripps Biomedical Research, 130 Scripps Way, Jupiter, 33458 Florida, United States; § Department of Chemistry, UF Scripps Biomedical Research, 130 Scripps Way, Jupiter, 33458 Florida, United States; ∥ Skaggs Graduate School of Chemical and Biological Sciences, The Scripps Research Institute, Jupiter, 33458 Florida, United States

**Keywords:** Lipid nanoparticles, mRNA, endosomal recycling, cellular uptake, mRNA delivery

## Abstract

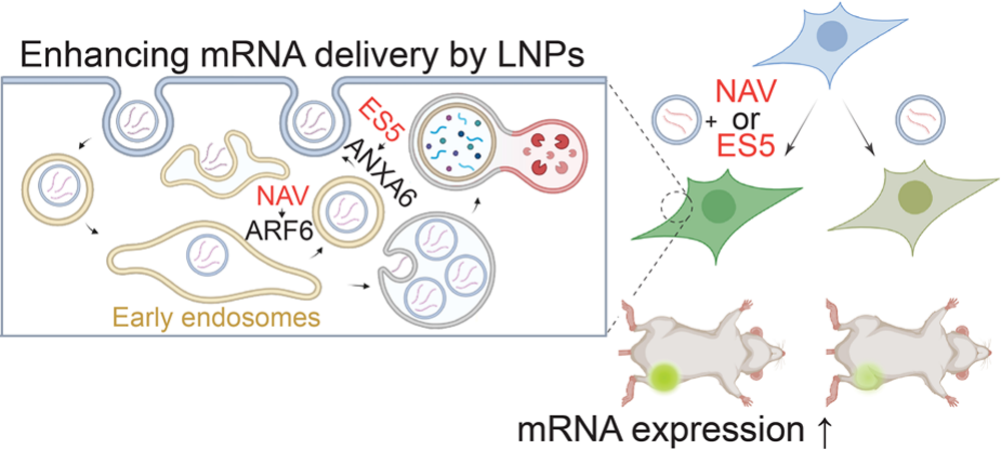

mRNA lipid nanoparticles (LNPs) have emerged as powerful modalities for gene therapies to control cancer and infectious and immune diseases. Despite the escalating interest in mRNA-LNPs over the past few decades, endosomal entrapment of delivered mRNAs vastly impedes therapeutic developments. In addition, the molecular mechanism of LNP-mediated mRNA delivery is poorly understood to guide further improvement through rational design. To tackle these challenges, we characterized LNP-mediated mRNA delivery using a library of small molecules targeting endosomal trafficking. We found that the expression of delivered mRNAs is greatly enhanced via inhibition of endocytic recycling in cells and in live mice. One of the most potent small molecules, endosidine 5 (ES5), interferes with recycling endosomes through Annexin A6, thereby promoting the release and expression of mRNA into the cytoplasm. Together, these findings suggest that targeting endosomal trafficking with small molecules is a viable strategy to potentiate the efficacy of mRNA-LNPs.

Over the past two decades, mRNA-LNPs have shown incredible performance for therapeutics development, as evidenced in the COVID-19 pandemic.^1^ Through LNP-mediated delivery, mRNAs encoding the designed therapeutic proteins can be rapidly expressed in the targeted cells and tissues to control a variety of human diseases, ranging from viral infection to cancer, immune, and neurological disorders. Moreover, recent studies have also succeeded in leveraging the robust expression of mRNA-LNPs with genome editing to reprogram T cells for developing powerful immunotherapies.^2^

The key to effective mRNA therapeutics lies in two fronts: the delivery and the translation of mRNAs in the targeted cells. Pioneering studies found that *in vitro* transcribed mRNAs readily activate dendritic cells *in vivo* and are quickly degraded by nucleases. Because of this challenge, initial efforts for the development of mRNA therapeutics were notoriously limited by the scant expression of the encoded proteins. The breakthrough came in with the discovery of pseudouridine that enabled the escape of delivered mRNAs from the innate immune system,^3^ thereby greatly boosting the efficacy of mRNA-LNPs. Further enhancements are also on the way with the recent development of self-replicating and circular mRNAs.^1^ Despite this progress, the delivery efficiency of mRNA remains low. It is estimated that only a small fraction of mRNAs encapsulated in LNPs were released into the cytoplasm and expressed. This limitation often necessitates high doses for each treatment and is now the main barrier to unleashing the full potential of mRNA therapeutics.

In order to augment the delivery of mRNA-LNPs, it is essential to understand how LNPs facilitate the entry of mRNAs into cells. Unfortunately, little is known on this front. LNPs were initially designed for the delivery of siRNAs.^1^ Through years of extensive research, the consensus is that LNPs promote the entry of siRNAs into cells through endosomal escape from prelysosomal compartments.^4^ Specifically, previous studies demonstrate that LNP uptake into cells is through endocytosis, during which the pH drops to around 5.5 in late endosomes. Taking advantage of these findings, current LNPs are engineered with carefully designed ionizable lipids that are largely neutral at pH 7 and become positively charged in acidic environments as in the endocytic pathway. In doing so, these positively charged ionizable lipids of LNPs rapidly engage with the negatively charged lipids of late endosomes, culminating in membrane fusion to enable the release of the siRNAs encased in LNPs. By virtue of the similar chemical compositions of siRNA and mRNA, it is thus proposed that mRNA follows the same delivery route via the fusion of LNPs with late endosomes.^1^ However, a recent study challenged this model by showing that mRNAs were released at the early stage of endosomal recycling.^5^

Thus, the molecular mechanism of LNP-facilitated mRNA delivery remains elusive, which limits further improvements in therapeutic developments based on this powerful approach. To bypass these limitations, we set out to screen small molecules that can boost mRNA-LNP delivery. In particular, many small molecules have already been developed to interfere with different steps of membrane trafficking and sensitize cells to siRNA therapeutics. In this manuscript, we assessed the impact of these small molecules on the efficacy of mRNA-LNPs in cells and in live mice. The results suggest that the endosomal recycling pathway is a critical target for research and therapeutic applications of mRNA-LNPs.

Extensive studies in the past two decades have generated a panel of small molecules that can manipulate almost every step during endosomal trafficking.^6−9^ Interestingly, a few of these small molecules are also known to enhance the delivery efficiency of siRNAs. Inspired by these studies, we set out to characterize the delivery of mRNA-LNPs in cells using a collection of small molecules targeting distinct stages of membrane trafficking during endosome biogenesis (Figure 1A). To enable rapid high-throughput screens and quantitative analysis, we encapsulated *in vitro* transcribed firefly luciferase (Fluc) mRNAs (Figure S1) into LNPs formed with a lipid mixture that is used in the current COVID-19 vaccines (50% SM102, 10% DSPC, 38.5% Cholesterol, 1.5% PEG2000-DSPE). These LNPs are 77 ± 2 nm in diameter by DLS measurements, and the encapsulation efficiencies are above 90% using ribogreen assays (Figure S2). The effect of small molecules on the expression of Fluc was quantified after 24-h delivery of mRNA-LNPs into HEK293T cells.

**Figure 1:**
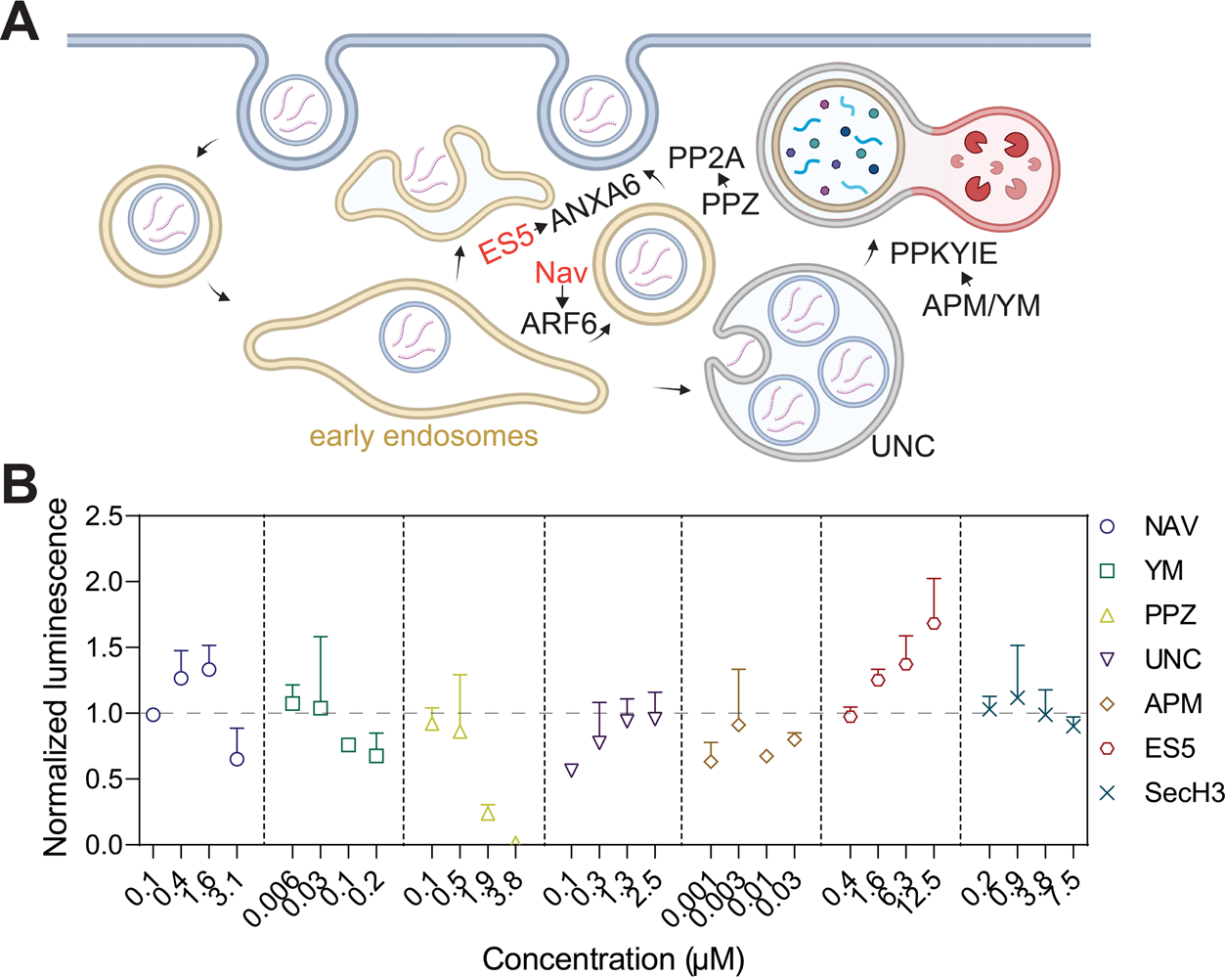
**Expression of mRNA-LNPs is enhanced by small molecules targeting recycling endosomes**. (**A**) A schematic representation of LNP-mediated mRNA release through endosomal trafficking and potential targets of small molecules characterized in this study. Created using BioRender.com. (**B**) Assessment of small molecules on the expression of LNPs harboring Fluc mRNAs in HEK293T cells. Data obtained with small molecules at the indicated concentrations were normalized to control experiments carried out with mRNA-LNPs alone (Dotted line). Mean values and standard deviations are indicated (*n* = 3).

To our surprise, many small molecules that are known to enhance endosomal escape of siRNA therapeutics, exhibited either little or inhibitory effects on mRNA-LNPs at concentrations that are still nontoxic to cells (Figure 1B and Figure S3A). Thus, the molecular mechanism for mRNA release might be distinct from that of siRNA. Nevertheless, we consistently found that two small molecules, NAV2729 (NAV) and endosidin 5 (ES5), resulted in significant enhancement (1.5–2 folds) of LNP-mediated delivery of Fluc mRNAs (Figure 1B and Figure S4). Incubation of NAV and ES5 together caused modest further increases in Fluc expression in comparison to the sole application of either compound (Figure S4); however, the effect of NAV and ES5 was not synergistic, indicating that they are most likely targeting the same membrane trafficking pathway. In addition, we also assayed the impact of the two compounds on siRNA delivery. Consistent with previous studies,^7^ NAV enhanced the silencing efficiency of siRNA, whereas ES5 exhibited insignificant effects (Figure S5). Hence, the delivery of siRNA and mRNA might not follow the same route in cells. Nevertheless, these findings suggest that the efficacy of mRNA-LNPs can be enhanced with small molecules. Moreover, the discovery of NAV and ES5 as chemical potentiators for mRNA-LNPs prompts us to investigate the underlying molecular mechanism.

Several possibilities can explain the increased delivery efficiencies of mRNA-LNPs. For example, it could be a result of the accelerated LNP uptake through endocytic pathways or the enhanced release of mRNA into the cytoplasm. To dissect these possibilities, we prepared two sets of LNPs: one set of LNPs contains the lipidic dye DiI C18 for direct analysis of uptake, and the other encapsulates GFP mRNAs to assess release efficiencies. We then used flow cytometry to characterize both the uptake of DiI C18-labeled LNPs and the expression of GFP mRNAs (Figure 2A-D). The results showed that NAV and ES5 had negligible effect on the uptake of DiI C18-labeled LNPs. In contrast, they both exhibited a ∼ 2-fold increase in the expression of GFP mRNAs, while the function of other types of small molecules were either trivial or inhibitory, in agreement with the data obtained using Fluc mRNA-LNPs (Figures 1B and S6). Again, cotreatment of NAV and ES5 together exhibited an accumulative but not synergistic effect of GFP mRNA delivery, supporting that they are regulating the same pathway during the release of mRNA-LNPs (Figure S7). Furthermore, we noticed that the effect of NAV showed a bell-shaped dose response in potentiating the delivery of mRNA-LNPs. The stimulatory effect by NAV peaked around 0.8–1.6 μM and started to diminish at higher concentrations. However, we did not detect much toxicity of either NAV or ES5 in cells (Figure S3B). Thus, we suspect that the decreased enhancement at higher concentrations of NAV might be related to its complex target profile.^10^

**Figure 2:**
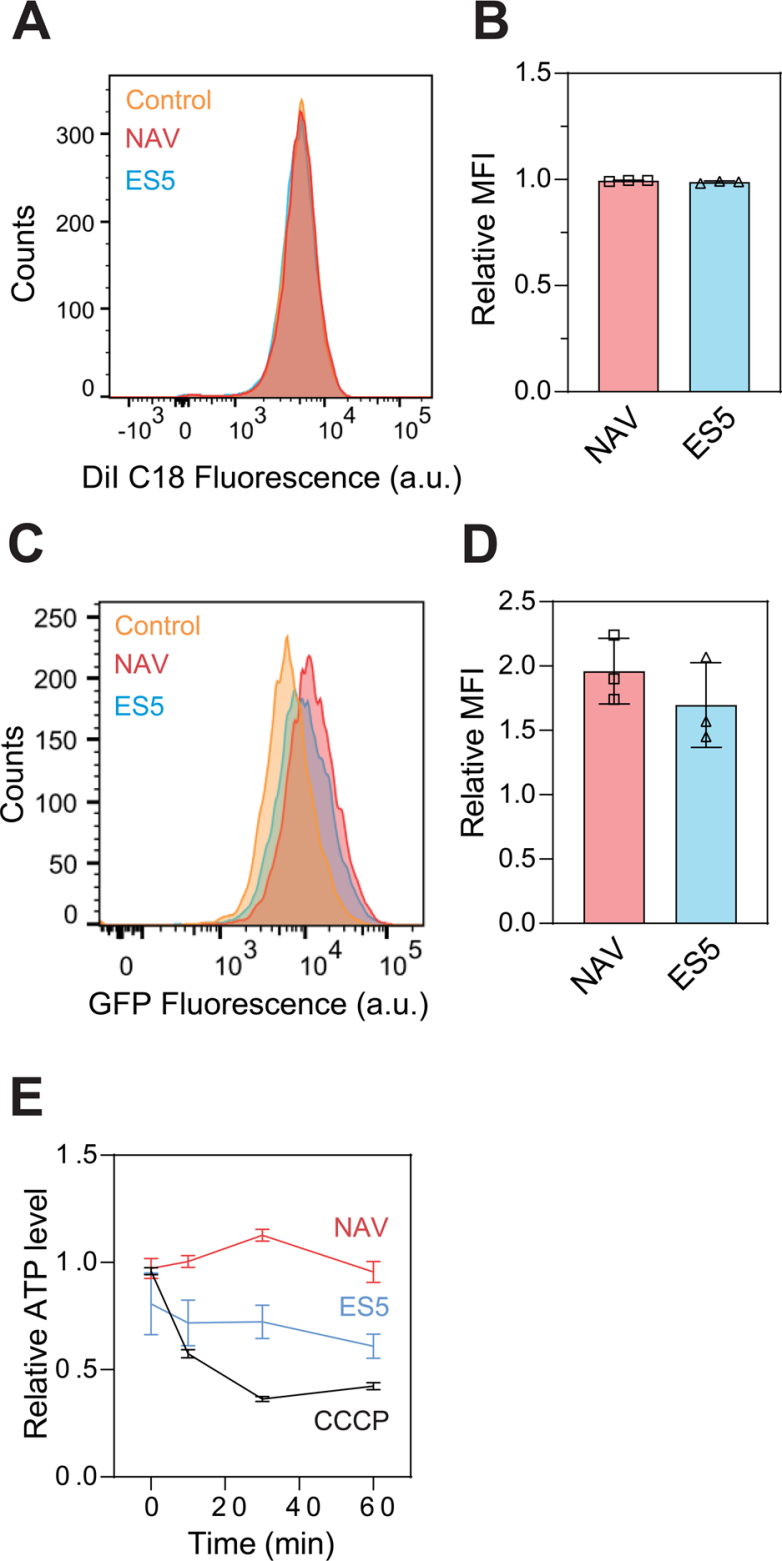
**NAV and ES5 enhance mRNA release from early endosomes**. (**A-B**) Representative flow cytometry histograms (A) and Mean Fluorescence Intensities (MFI) (B) of HEK293T cells treated with LNPs labeled with DiI, in the absence (control) or presence of 1.6 μM NAV or 6.3 μM ES5. (**C–D**) Representative flow cytometry histograms (C) and MFI (D) of HEK293T cells treated with LNPs harboring GFP mRNAs, in the absence (control) or presence of NAV or ES5. In panels B and D, MFI of cells in the presence of 1.6 μM NAV or 6.3 μM ES5 was normalized to control cells. (**E**) Cellular ATP levels after treatments with the indicated small molecules. Expeiments were carried out using 1.6 μM NAV, 6.3 μM ES5, and 10 μM CCCP. Data were collected from three independent experiments and are shown as mean ± s.d.

Our data are insufficient to determine the rate-limiting step of LNP-mediated mRNA delivery. Addressing this question will require more rigorous kinetic measurements of mRNA uptake and release. Based on our findings, we suggest that uptake and release of mRNA-LNPs can be separately manipulated with small molecules. If the uptake of mRNA-LNPs is indeed the rate-limiting step, future work should certainly explore small molecules to boost this step and use them together with NAV/ES5 for mRNA delivery.

Furthermore, it is possible that NAV and ES5 enhanced the efficacy of mRNA-LNPs by targeting metabolic pathways, thereby interfering with cellular processes that are energy-dependent, such as clathrin-mediated endocytosis. To test this idea, we assessed if NAV and ES5 would modulate ATP concentrations in cells. Interestingly, the results showed that ATP levels were unaffected by NAV, but considerably decreased by ES5 (Figure 2E). This effect of ES5 could be due to a protonophore activity as observed in other endosidin small molecules,^11,12^ suggesting that additional optimization is needed to increase the specificity of this compound for potentiating mRNA delivery. Nevertheless, the decrease in cellular ATP levels should only exhibit inhibitory effects on the uptake of mRNA-LNPs. Therefore, we concluded that the observed effects of NAV and ES5 were a direct manifestation of increased mRNA release during endosomal trafficking.

Both NAV and ES5 target recycling endosomes. NAV is a potent inhibitor for ARF6-dependent endocytic recycling,^13^ whereas ES5 is identified from a high-throughput screen on membrane trafficking.^6^ The molecular target of ES5 is unclear, but it is known to promote the formation of tubular endosomal networks. Thus, we performed a cellular thermal shift assay (CETSA) in conjunction with quantitative mass spectrometry to probe the potential target of ES5.^14^ Cell lysates were subjected to a temperature gradient in the presence of ES5 or DMSO before pelleting insoluble debris. The soluble proteome was subsequently processed for quantitative proteomics to reveal ligand specific changes in protein stability. In comparison with ES5-treated samples, Annexin A6 (ANXA6) was markedly enriched in samples treated with DMSO alone (Figure 3A). This observation was further validated by Western blot (Figure 3B) using a monoclonal antibody against ANXA6.

**Figure 3:**
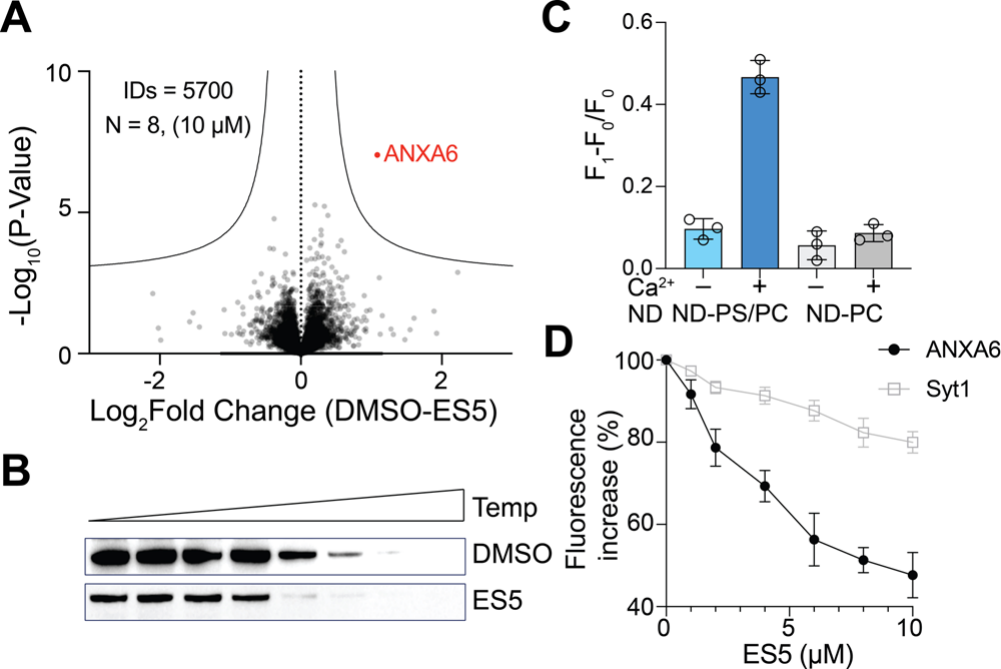
**ES5 inhibits ANXA6-lipid interactions**. (A) HEK293T cell lysates were treated with 10 μM ES5 or DMSO and analyzed by CETSA on a thermal gradient from 38 °C - 61 °C. Chemoproteomics results are represented as a volcano plot of -log_10_(p-value) vs Log_2_(DMSO-ES5). *N* = 8 for each treatment. FDR of 0.05 is indicated by the black dotted line. (B) Western blot of CETSA soluble fractions across temperature gradient of 38 °C - 61 °C stained for ANXA6, validating that ANXA6 stability is altered by ES5 treatment. (C) Probing ANXA6 binding to lipids using a membrane nanodisc (ND) reporter.^15^ Fluorescence of the ND sensor reconstituted with either PC or PS/PC lipids was quantified before (F0) and after (F1) the addition of ANXA6. (**D**) Membrane binding and remodeling of ANXA6, but not synaptotagmin-1 (syt1), is drastically inhibited in the presence of increasing amounts of ES5. Mean values and standard deviations are indicated (*n* = 3).

We then characterized the interaction of ES5 with ANXA6 in reconstituted systems. ANXA6 is a Ca^2+^-dependent lipid-binding protein that mediates membrane tethering and remodeling in the endocytic pathway. Hence, we suspected that ES5 disturbs the interaction of ANXA6 with lipids. To test this hypothesis, we assayed the impact of ES5 on ANXA6-lipid interactions using a robust membrane remodeling sensor developed in our recent work.^15^ In the absence of ES5, ANXA6-mediated membrane remodeling was readily detected and required Ca^2+^ and anionic lipids (Figure 3C), consistent with previous studies.^16^ However, this activity was drastically inhibited by ES5 in a concentration-dependent manner with an EC_50_ of ∼3 μM (Figure 3D). As a control experiment, we also assayed the impact of ES5 on another Ca^2+^-dependent lipid binding protein, synaptotagmin-1 (syt1), the Ca^2+^ sensor for neurotransmission. The results showed that ES5 only marginally repressed the membrane remodeling activity of syt1. Thus, ES5 is a potent and specific inhibitor of ANXA6-lipid interactions.

Next, we investigated the role of ANXA6 in the ES5-enhanced delivery of mRNA-LNPs in cells. For this purpose, we attempted to knockout (KO) ANXA6 using Cas9-CRISPR and cognate gRNAs. Among different designs of gRNAs (Table S1), gRNA4 showed the highest KO efficiency (Figure 4A), whereas modest levels of ANXA6 expression were still observed with gRNA2 and gRNA3. With these gRNAs in hand, we then assayed the correlation of ANXA6 KO with ES5-enhanced delivery of mRNA-LNPs in cells. The results showed that the enhancement of mRNA-LNP delivery by ES5 was considerably decreased in cells treated with gRNA4 (Figure 4B). In contrast, insufficient KO of ANXA6 with gRNA2 and gRNA3 did not bring about significant changes in mRNA-LNP delivery than control experiments performed with nontargeting gRNA1. These observations were accompanied by the increased delivery of mRNA in the absence of ES5 in these KO cells due to the loss of ANXA6 (Figure S8A). In addition, the specificity of ES5 for ANXA6 is further supported by the finding that the stimulatory function of NAV was not affected in ANXA6 KO cells (Figure S8B). Thus, these data are in line with our observations from *in vitro* experiments (Figure 3), suggesting that ES5 enhances LNP-mediated mRNA delivery by blocking ANXA6-lipid interactions at the early stage of endocytic recycling. However, the underlying molecular mechanism remains elusive. ANXA6 is a well-established regulator of endocytic pathways, governing clathrin-coated-pit budding events and membrane organization.^16^ Recent studies revealed the role of ANXA6 in coupling membrane repair with exosome release.^17^ It is proposed that ANXA6 tethers membranes and regulates vesicular transport through its lipid binding activities upon Ca^2+^ influx. Thus, we posit that inhibition of ANXA6-lipid interactions might promote mRNA release from tubular recycling endosomes and/or prevent the clearance of mRNA-LNPs through the exocytic pathway.

**Figure 4:**
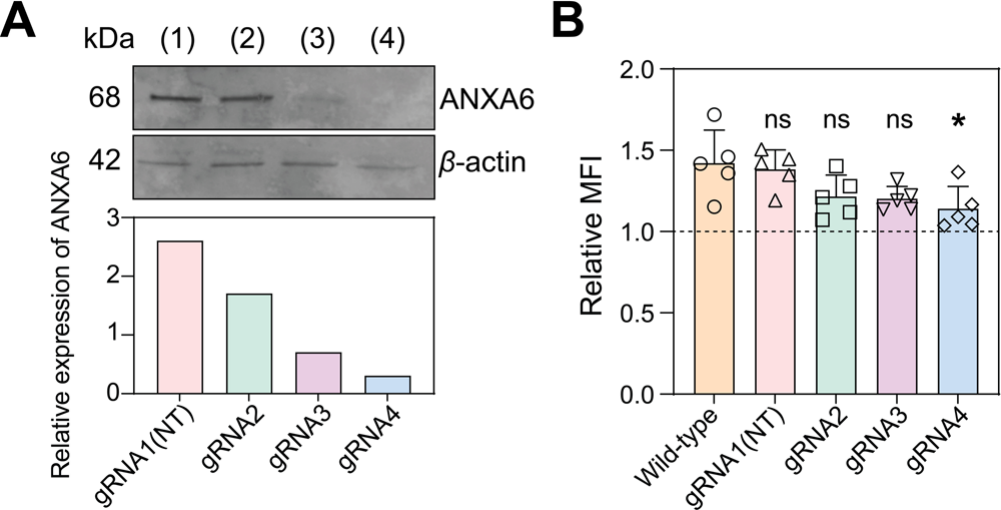
**The stimulatory effect of ES5 is mediated by ANXA6 in cells**. (**A**) Analysis of the knockout (KO) efficiency of ANXA6 in HEK293T cells by immunoblot. One nontargeting (NT) (1) and three specific gRNAs (2–4) were assayed for ANXA6 KO using CRISPR-Cas9. The expression levels of ANXA6 relative to *ß*-actin were quantified at each condition. (**B**) Characterization of ES5-stimulated expression of GFP mRNA-LNPs in HEK293T cells treated with CRISPR-Cas9 and the indicated gRNAs. Five LNP delivery experiments were performed with two independent preparations of ANXA6 KO cells. Statistics of relative fluorescence intensities per each treatment were compared to a control group (wild type) using unpaired two-tailed Student’s *t* test (ns = not significant; * = *p* < 0.05). Data are shown as mean ± s.d.

To further understand the molecular mechanism of ES5 and NAV, we also determined the impact of these two compounds in ARF6 KO cells. We did observe that NAV was no longer able to enhance the efficacy of mRNA-LNPs in ARF6 KO cells (Figure S9). However, we also found that ARF6 KO decreased mRNA delivery in the presence of ES5, most likely because of the multifaceted function of ARF6 in membrane trafficking.

Encouraged by the effects of NAV and ES5 on mRNA-LNPs in cell-based experiments, we further characterized these two molecules *in vivo* (Figure 5). To this end, we incubated NAV or ES5 with Fluc mRNA-LNPs and performed intramuscular (IM) injections in mice. The expression of Fluc was quantified using bioluminescence imaging (Figure 5A and B). We found that NAV significantly enhanced the delivery efficiency of Fluc mRNA-LNPs (Figure 5B and C). The stimulatory effect of NAV *in vivo* requires much higher concentrations than that of *in vitro* assays, indicating that the difference in cell types and local environment are important parameters modulating the efficacy of mRNA-LNPs (Figure S10). Furthermore, the complex target profile of NAV might also limit its efficacy at high concentrations used for *in vivo* experiments. Although the effect is less potent than our findings obtained from cell-based assay, it is significant and suggests that targeting endosomal recycling is a viable strategy to enhance LNP-mediated mRNA delivery *in vivo*.

**Figure 5:**
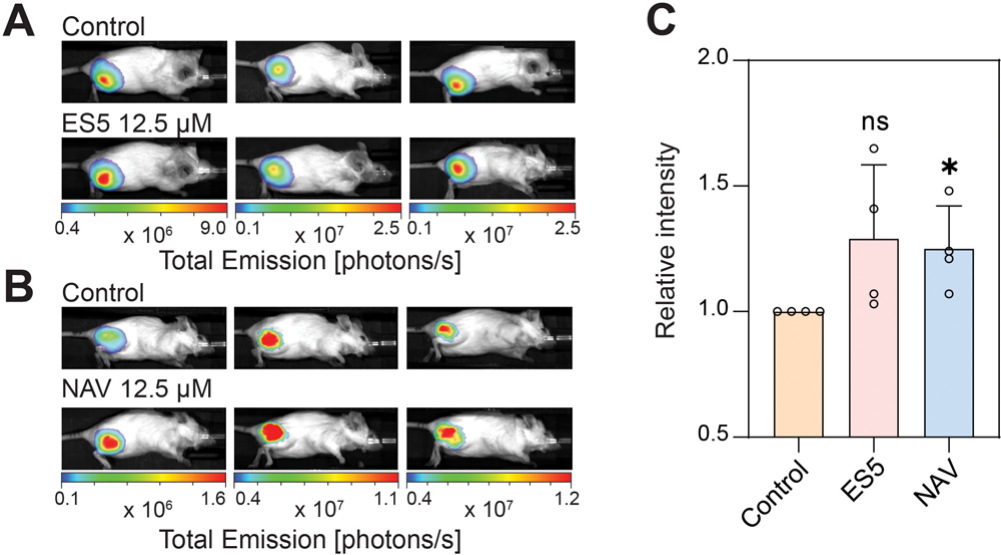
**Characterization of NAV and ES5*****in vivo***. (**A-B**) Representative images of Balb/C mice that are intramuscularly (IM) injected with Fluc mRNA-LNPs along with 12.5 μM of ES5 (A) and 12.5 μM of NAV (B). Bioluminescence was quantified 24 h postinjection. Four independent experiments (*n* = 2–3 per group) were carried out. (**C**) Bioluminescence of Fluc mRNA expression quantified from panels A and B were normalized to controls. Statistics of relative bioluminescence intensities per each treatment were analyzed using unpaired two-tailed Student’s *t* test (ns = not significant; * = *p* < 0.05). Data are shown as mean ± s.d.

In contrast, we did not observe much effect of ES5 (Figure 5A), probably because its protonophore activity impeded the delivery of mRNA-LNP *in vivo*. In addition, we injected small molecules together with mRNA-LNPs to simplify the administration strategy for *in vivo* experiments. This approach is not optimal as NAV and ES5 only have a very short time window to exert any effects. Thus, we had to use much higher concentrations for *in vivo* experiments than *in vitro* assays, in which we pretreated cells beforehand. To further boost the efficacy of these compounds for enhancing mRNA delivery *in vivo*, future work should focus on screening and optimizing administration routes. Nevertheless, the enhancement by NAV is consistent with our *in vitro* results and supports that the endosomal recycling pathway is a focal point for potentiating the efficacy of mRNA-LNPs in cells and in animal models.

Together, our data suggests that LNP-mediated delivery of mRNA is different from siRNA. Several small molecules that sensitize cells to siRNA therapeutics are not effective for mRNA-LNPs. These molecules (e.g., UNC and YM) inhibit the late stage of endosomal trafficking (Figure 1A) and enhance the escape of siRNA during the biogenesis of MVBs and autophagosomes. Since a previous study also indicated that mRNAs were released at the early stage of endosomal recycling,^5^ it is thus not surprising that reagents targeting late endosomes are not exhibiting any effect on mRNA-LNPs. Consistently, we only observed stimulating effects by NAV and ES5 that both act on endocytic recycling. Thus, delivery of mRNAs by LNPs is distinct from that of siRNAs and most likely occurs through early recycling endosomes.

A number of possibilities can explain these differences. mRNAs are much larger molecules than siRNAs, and their release through membrane fusion might require rapid expansion of fusion pores that only occurs during tubular recycling endosome biogenesis. Moreover, mRNAs carry much more negative charges, and their binding to lipids might be too tight to be released as compared to siRNAs in late endosomes. As such, they can only be released in less acidic environments as in early endosomes. Finally, mRNAs are packed differently in LNPs,^18,19^ making them unable to escape during vesiculation in prelysosomal compartments.

Regardless of the potential mechanism underlying the difference between siRNA and mRNA delivery by LNPs, our work, together with previous studies, showcases the utility of small molecules to dissect the biology of endosomal trafficking for therapeutical applications of nucleotide drugs. For example, an elegant work by Finicle et al. developed a potent compound, SH-BC-893, that vastly improved the performance of siRNA therapeutics in animal models.^7^ Despite the difference in the release sites of mRNAs and siRNAs, inhibition of endocytic recycling greatly improves their therapeutic efficacies. Armed with powerful small molecules targeting endosomal trafficking, we expect that advanced imaging approaches will provide in-depth mechanistic insights into basic and translational studies of oligonucleotide therapeutics.

Herein, we report that NAV and ES5 enhance the efficacy of mRNA-LNPs in cells and in live mice by inhibiting endocytic recycling. We screened a panel of chemical modulators of the endocytic pathways and identified two molecules, NAV and ES5, that increased the delivery efficiencies of mRNA-LNPs *in vitro* and *in vivo*. To dissect the underlying mechanism, we characterized the function of these two small molecules in the biology of mRNA-LNPs. The results suggested that NAV and ES5 do not play a role in accelerating mRNA-LNP uptake nor boosting cell metabolism, but they rather target recycling endosomes to promote mRNA release. NAV blocks the activation of the essential regulator of endosomal trafficking, ARF-6 (ref (7).) and thus inhibits endocytic recycling. ES5, on the other hand, suppresses the function of ANXA6 during early endosome biogenesis. We uncover that ES5 binds ANXA6 and obstructs the interaction of ANXA6 with membranes, thereby facilitating mRNA-LNP delivery. This is further supported by data obtained using ANXA6 KO cells in which the ES5-enhanced delivery of mRNAs by LNPs is significantly reduced. We propose that ES5-mediated inhibition of ANXA6 potentiates mRNA-LNPs by regulating the remodeling and/or the exocytosis of early endosomes. Despite engaging different targets, the function of NAV and ES5 converge on endocytic recycling to augment the delivery of mRNA-LNPs. Moreover, the finding that recycling endosomes might be the site for mRNA-LNP release calls for further optimization of ionizable lipids to target the early stage of endocytosis. The lipid compositions of early endosomes are very different and might require screening other types of structural lipids for mRNA release. Potent small molecules to manipulate mRNA-LNP biology will serve as useful tools for these purposes, thereby expediting the development of next-generation nucleic acid therapeutics.

## Supporting Information Available

Detailed experimental procedures for mRNA containing lipid nanoparticle formulation and characterization, mRNA lipid nanoparticle delivery *in vitro* and *in vivo*, Annexin A6 and ARF6 knock out experiments (PDF)
