# Supplementary material for: Targeting Recycling Endosomes to Potentiate mRNA Lipid Nanoparticles
Source: Nano Lett. 2024 Apr 19;24(17):5104–9. doi: 10.1021/acs.nanolett.3c04415 (PMC11066955; doi:10.1021/acs.nanolett.3c04415)
Supplement: Supplementary file 1 — nl3c04415_si_001.pdf [file nl3c04415_si_001.pdf]

Supporting Information for

# Targeting recycling endosomes to potentiate mRNA lipid nanoparticles

Jeehae Shin[a,b], Cameron J. Douglas[c,d], Shanwen Zhang[b], Ciaran P. Seath[c,d], and Huan Bao[a,b†]

[a]Department of Molecular Physiology and Biological Physics, University of Virginia, 480 Ray C. Hunt Drive, Charlottesville, 22903, Virginia, USA

[b]Department of Molecular Medicine, UF Scripps Biomedical Research, 130 Scripps Way, Jupiter, 33458, Florida, USA

[c]Department of Chemistry, UF Scripps Biomedical Research, 130 Scripps Way, Jupiter, 33458, Florida, USA.

[d]Skaggs Graduate School of Chemical and Biological Sciences, The Scripps Research Institute, Jupiter, 33458, Florida, USA.

†Corresponding author: E-mail: baoh@virginia.edu

**Table of Contents**

1. Experimental section ........ S2

2. Validation of *in vitro* transcribed mRNA and LNP formulation ........ S12

3. Small molecule treatment on 293T cells ........ S13

4. Sequence information ........ S19

## 1. Experimental section

### 1.1 General chemicals

1,2-distearoyl-sn-glycero-3-phosphocholine (DSPC) (850365),1,2-dimyristoyl-sn-glycero-3-phosphoethanolamine-N-[methoxy(polyethyleneglycol)-2000] (ammonium salt) (PE-PEG 2k) (880150) and cholesterol (700100) were purchased from the Avanti®. 1,1'-Dioctadecyl-3,3,3',3'-Tetramethylindocarbocyanine Perchlorate (DiIC18) was obtained from Invitrogen (D3911). SM102 was purchased from Astatech (E77307). Small molecules were obtained from various sources: Endosidin 5 (ES5) (Cambridge Corporation, 5522797), NAV 2729 (NAV) (Tocris Bioscience, 5986), YM201636 (YM) (Apexbio Technology LLC, B21895), perphenazine (PPZ) (TCI America, P1970), UNC10217938A (UNC) (MedChemExpress LLC, HY-136151), Apilimod (APM) (MedChemExpress LLC, HY-14644), SecinH3 (SecH3) (Medchemexpress LLC, HY-100559). Unless otherwise specified, all other chemicals were purchased from Merck and Sigma.

### 1.2 Methods

#### 1.2.1 mRNA *in vitro* transcription and encapsulation in lipid nanoparticles

Plasmids encoding green fluorescent protein (GFP) and firefly luciferase (Fluc) for mRNA transcription *in vitro* were gifts from Drs. Michael Farzan and Hyeryun Choe. Both plasmids were made based on the pUC-ccTEV-A101 vector containing T7 promoter, 5'UTR, 3' UTR, and poly A tail. mRNAs were transcribed from linearized plasmids *in vitro* using MEGAScript T7 kits (Invitrogen, AMB13345). UTP was replaced with m1Ψ-5'-triphosphate (TriLink, N-1081) and 5'cap was added using CleanCap (TriLink, N-7113) during the transcription process. The synthesized mRNAs were analyzed by a denaturing agarose gel. mRNA strands were further purified by cellulose columns and stored at -20 °C. mRNAs were encapsulated into lipid nanoparticles (LNPs) using Ignite Nanoassemblr (Precision Nano Systems). Specifically, mRNAs were prepared in 25mM sodium acetate buffer (pH 5.5) and lipids consisted of SM102, DSPC, cholesterol, PE-PEG 2k in a molar ratio of 50:10:38.5:1.5 were dissolved in ethanol. mRNAs and lipids at an N/P ratio of 4 were then rapidly mixed by Ignite Nanoassemblr at a

total flow rate of 6 mL/min and a ratio of 3:1 (aqueous phase: organic phase). The formulated mixture was then dialyzed against PBS for 24 hours and the buffer was refreshed three times. After dialysis, mRNA-LNPs were analyzed by dynamic light scattering (DLS) and further concentrated by centrifugal filter (Amicon, 30,000 Da MWCO) at 3,700 rpm for 15 minutes at 4°C. The quantity of mRNAs encased in LNPs was determined by ribogreen assay (Thermo Fisher Scientific, R11490). For short-term storage (within a month), LNPs were stored at 4°C. For long-term storage, LNPs were mixed with sucrose at a final concentration of 10% and stored at -80 °C.

### 1.2.2 Small molecule treatment and mRNA-LNP delivery to HEK293T cells

HEK293T cells were cultured at 37°C with 5% $CO_2$. Cells were maintained in Dulbecco's Modified Eagle's Medium (DMEM) (Thermo Fisher Scientific, 10569044) supplemented with 10% fetal bovine serum (FBS) (Thermo Fisher Scientific, 16000044) and 1x penicillin-Streptomycin (pen/strep) (Thermo Fisher Scientific, 15140122). Prior to transfection, cells were seeded on 96- or 48-well plates at its 40% confluency using complete media without pen/strep. After 16-18 hours, culture media was replaced with media containing each small molecule. For controls, media without small molecules were applied. The following stock solutions were prepared in DMSO: 50 mM ES5, 12.5 mM NAV, 0.8 mM YM, 15 mM PPZ, 10 mM UNC, 0.1 mM APM, and 30 mM SecH3. These small molecules were then diluted in the culture media to the indicated concentrations and applied to cells. After treatments with small molecules, 125 or 250 ng mRNA-LNPs (GFP or Fluc) were added to cells grown on 96- or 48-well plates, respectively. Samples were further incubated for 20-24 hours before analysis.

### 1.2.3 Quantification of luciferase expression

Fluc mRNA delivery by LNPs was conducted using cells grown on 96-well plates. After treatment of mRNA-LNPs for 20-24 hours, cells were washed with cold PBS and subjected to analysis using a luciferase assay Kit (Genecopoeia, LF009). Specifically, cells were first treated with 40 µl of lysis buffer and the lysates were transferred to 96-well white plates. Next, 100 µl of reaction buffer was added using a multi-channel pipette to each sample.

Luminescence signals were then measured by a Synergy H1M plate-reader. Data points were plotted using GraphPad.

### 1.2.4 Quantification of GFP expression

GFP mRNA delivery was carried out on 96-well plates for dose-response experiments of small molecules. For others experiments, GFP mRNA delivery was carried out on 48-well plates. After 20-24 hours of GFP mRNA-LNP delivery, cells were washed with cold PBS and trypsinized at 37°C. Trypsin was deactivated by adding complete media without pen/strep. The resulting cell suspension was filtrated through a cell strainer and run through a flow cytometer for GFP quantification. The cells cultured on 96-well plates were analyzed by Guava easyCyte 8HT (Cytek BioSciences), and the cells cultured on 48-well plates were analyzed by LSRII (BD Bioscience). Flow cytometry data were analyzed using FlowJo and data points were plotted using GraphPad Prism 10.

### 1.2.5 Cell viability test

HEK 293T cells were seeded at 40% confluency on 96-well plates and incubated overnight. The media was refreshed with media containing the respective concentrations of small molecules. In control experiments, media without small molecules was used. After incubation for 24 hours, cells were washed with cold PBS, trypsinized at 37°C and resuspended in PBS for assessment using LIVE/DEAD™ Cell Vitality Assay Kit (Thermo Fisher Scientific, L34951) and Guava easyCyte 8HT.

### 1.2.6 Measurement of ATP levels

HEK293T cells were seeded in tissue-culture treated, white 96-well plates at its 40% confluency and incubated overnight. The culture media was replaced with the media containing the respective small molecules at the indicated concentrations. Media without small molecules was used as controls. After media replacement, a reaction buffer of ATPlite 1step Luminescence Assay System (Perkin Elmer, E77307) was added to the well at a 1:1 (media: ATPlite solution) v/v ratio at each time point. After 3-minute incubation on an orbital shaker, luminescence was read by a microplate reader. Data points were plotted using GraphPad

Prism 10.

### 1.2.7 Preparation of DiIC18-labeled LNPs for delivery to HEK293T cells

DiIC18 dissolved in ethanol was added to the lipid mixture for preparing mRNA-LNPs at a molar ratio of 1:1200. DiIC18-labeled LNPs were prepared by rapid mixing of 25mM sodium acetate buffer with the lipid mixture containing DiIC18 in ethanol using Ignite Nanoassemblr and then delivered to HEK293T cells. Uptake of DiIC18-labeled LNPs into cells were characterized by flow cytometry. Data were analyzed and plotted using FlowJo and GraphPad Prism 10.

### 1.2.8 Unstable GFP (uGFP) KD by siRNA-LNPs

siRNA strands targeting GFP were purchased from IDT (51-01-05-07). Unstable GFP (uGFP) expressing HEK 293T cells (Gen Target Inc., SC058) were maintained in complete media with 1μg/mL of puromycin. For siRNA delivery, cells were seeded on 96-well plates at 40% confluency without puromycin and incubated overnight. Cell media were then replaced with media containing the respective concentrations of small molecules. siRNAs were delivered using a RNAiMAX Transfection Reagent (Thermo Fisher Scientific, 13778075) according to the manufacturer's protocol. After 48 hours, cells were washed with cold PBS, trypsinized, and redispersed in fresh media for analysis using Guava easyCyte 8HT.

### 1.2.9 Generation of ARF6 KO cells

HEK293T cells were seeded at 50% confluency on 48-well plates without pen-strep and incubated overnight. 200 ng of ARF6 CRISPR/Cas9 knock-out (KO) plasmids co-expressing gRNAs (Santa Cruz, sc-400799) were added to the cells using Lipofectamine™ 2000 Transfection Reagent (Thermo Fisher Scientific, 11668019). The cells were incubated for 48 hours and then used for mRNA delivery experiments.

### 1.2.10 Immunoblotting of ARF6 KO cells

Wild-type and ARF6 KO cells were washed in PBS and disrupted by resuspending in PBST containing 1% triton-X100 plus 1X protease inhibitor cocktail (Thermo Fisher Scientific, A32955) for 15 minutes on ice. The cell lysates were clarified by spinning down at 15,000 x g

for 10 minutes at 4°C and then mixed with SDS-PAGE loading buffer and heated to 95°C for 5 minutes. Samples were analyzed by a 4–20% gradient gel (Bio-rad, 4561096) and transferred to a PVDF membrane (Bio-rad, 1704156EDU). The membrane was blocked with 5% dry milk in TBST (0.1% tween 20) for 30 minutes and washed with TBST three times. To detect the KO efficiencies of ARF6, the membrane was incubated with 1:10 diluted primary antibodies against ARF6 (Santa Cruz, sc-797) in TBST containing 5% dry milk at 4°C overnight. After washing with TBST three times, the membrane was incubated with 1:200 diluted HRP-conjugated anti-mouse secondary antibodies (Santa Cruz, sc-516102) in TBST containing 5% dry milk for 1 hour at room temperature. After washing, the membrane was developed in Pierce™ ECL Western Blotting Substrate (Thermo Fisher Scientific, 32109) and visualized using Chemi-Doc (Bio-rad). For data normalization, the same membrane was washed with TBST and incubated with 1:200 diluted beta-actin (Santa Cruz, sc-47778 B) in TBST containing 5% dry milk at 4°C overnight. After washing the membrane with TBST for three times, 1:200 diluted HRP-conjugated anti-mouse secondary antibodies were added to the membrane and incubated at room temperature for an hour. The membrane was developed in ECL solution after washing with TBST, and visualized using Chemi-Doc. The corresponding intensities of each protein band were analyzed by ImageJ and plotted by GraphPad Prism 10.

#### 1.2.11 Small molecule treatment and mRNA-LNP delivery to ARF6 KO cells

ARF6 KO cells were seeded at 40% confluency on 48-well plates. After 16-18 hours, the ARF6 KO cells were refreshed with media in the absence of small molecules for control experiments or in the presence of small molecules (1.6 µM NAV and 6.3 µM ES5). Next, 250 ng of Fluc mRNA-LNP was delivered, and the cells were further incubated for 20-24 hours. Cells were then washed with cold PBS and treated with 80 µl of lysis buffer (Genecopoeia, LF009). Cells lysates (40 µl) were transferred to 96-well white plates and 100 µl of reaction buffer was added using a multi-channel pipette for quantification of luminescence by a microplate-reader. Data were plotted using GraphPad Prism 10.

#### 1.2.12 Generation of Annexin A6 KO cells

We utilized Alt-R S.p. Cas9 V3 (glycerol-free) system (IDT) to KO ANXA6 with three specific gRNAs and a non-targeting control as detailed in Table S1. Electroporation of ribonucleoprotein (RNP) complex was performed by following the protocol from IDT. First, equal volume of 200 μM gRNA and 200 μM tracrRNA were mixed and heated to 95°C for 5 minutes and slowly cooled down to 4°C. To form RNP complex, 4.8 μl of gRNA/tracrRNA, 8.6 μl PBS, and 6.8 μl of 62 μM Alt-R Cas9 enzyme were mixed and incubated at room temperature for 20 minutes. During RNP formation, HEK293T cells, in complete media without pen/strep, were trypsinized and redispersed in fresh media. After counting the number of viable cells, cell suspension was centrifuged down at 100 x g for 5-10 minutes at room temperature and washed with PBS. Nucleofection solution from SF Cell Line 4D-Nucleofector™ X Kit L (Lonza, V4XC-2012) was added to the cell pellet to yield 1.2-1.4E6 cells/80 μl. The pre-formed RNP complex and ssDNA enhancer were added to the cell solution and electroporation was carried out using a 4D-Nucleofector instrument (Lonza). Subsequently, pre-warmed complete media was added to the cells and transferred to a 6-well plate. After 24 hours of post-electroporation, media was refreshed and incubated for another day before analysis of KO efficiencies.

### 1.2.13 Immunoblotting for Annexin A6 KO.

Cell lysates were analyzed by denaturing gel electrophoresis and western blot as described in 1.2.9. The resulting membrane was then incubated with 1:100 diluted primary antibodies against ANXA6 (Santa Cruz, sc-271859) and beta-actin (Santa Cruz, sc-47778 B) in TBST containing 5% dry milk and 5% BSA at 4°C overnight. After washing with TBST three times, the membrane was incubated with 1:200 diluted FITC conjugated anti-mouse secondary antibodies (Santa Cruz, sc-516140) in TBST containing 5% dry milk for 1 hour at room temperature. After washing, the membrane was visualized using Chemi-Doc (Bio-rad). The intensities of ANXA6 and beta-actin bands were quantified by ImageJ and plotted by GraphPad Prism 10.

### 1.2.14 Small molecule treatment and mRNA-LNP delivery to ANXA6 KO cells

ANXA6 KO cells grown on 6-well plates were trypsinized, redispersed in a complete media for cell counting, and then seeded on 48-well plates at its 40% confluency. After overnight incubation, we treated the cells with small molecules and delivered 250 ng of GFP-LNPs. After 20-24 hours, cell suspensions were prepared and analyzed via flow cytometry. FlowJo and GraphPad Prism 10 were used for generating data plots and p-values.

### 1.2.15 Bioluminescence imaging with small molecules

Experimental procedures were approved by IACUC (protocol number: 22-002-01) and were followed by the guidelines. 12-week-old, female Balb/C strain mice (Jackson Laboratory) were used for *in vivo* studies. 1 µg Fluc-LNP was mixed with small molecules in PBS at the indicated concentrations in a total volume of 20 µl. As a control, the same Fluc-LNP (1 µg in 20 uL PBS) without small molecules was prepared. Injection was performed intramuscularly, and after 24 hours, 100 µl of RediJect solution (Perkin Elmer, 770505) was injected intraperitoneally to examine the magnitude of bioluminescence under Lago X imager. During the injection and bioluminescence imaging processes, the mice were anesthetized by isoflurane flow. The obtained data were processed using Aura; data points were plotted, and p-values were generated by GraphPad Prism 10.

### 1.2.16 CESTA

HEK293T cell pellets were lysed in reticulocyte standard buffer containing 10mM Tris-HCl, 15mM NaCl, 1.5mM $MgCl_2$, and 1x Halt protease inhibitor cocktail (Thermo Fisher Scientific, 78438). Lysates were incubated on ice for 20 minutes followed by clarification via centrifugation at 17,000 x g for 30 minutes at 4°C. The soluble fraction was transferred to a microcentrifuge tube and the protein concentration was measured by Pierce BCA protein assay (Thermo Fisher Scientific, 23225). Cell lysates were diluted to 1.5 mg/mL in a lysis buffer and allowed to warm to room temperature. Subsequently, they were treated with either DMSO or 10 µM ES5 in DMSO and allowed to incubate for 15 minutes at room temperature. Each treatment was then divided into 8 fractions in 200µl PCR strip tubes and heated across

a temperature gradient from 38°C to 61°C for 3 minutes in a thermocycler. Each fraction was allowed to cool to room temperature and transferred to a 1.5 mL microcentrifuge tube. Precipitated proteins were pelleted by centrifugation at 17,000 x g for 15 minutes. The soluble fractions were transferred to fresh 1.5 mL microcentrifuge tubes.

An aliquot of each fraction was run on a NuPAGE 4-12% Bis-Tris acrylamide gel (Thermo Fisher Scientific, NP0321BOX) and transferred to a nitrocellulose membrane. The membrane was incubated overnight at 4°C with primary antibodies for ANXA6 (Abcam, ab201024) diluted by 1:1000 in TBST, then washed with TBST. Subsequently, we treated the membrane with secondary goat anti-rabbit HRP conjugated antibodies (ImmunoReagents, GtxRb-003-DHRPX) diluted by 1:10,000 in TBST and incubated them for 1 hour at room temperature. The membrane was washed with TBST and then briefly incubated with SuperSignal™ West Pico PLUS Chemiluminescent Substrate (Thermo Fisher Scientific Scientific, 34580) and imaged.

**1.2.17 LC/MS sample preparation**

20 µg of proteins from each CETSA fraction were prepared for LC/MS analysis using Sera-Mag Carboxylate SpeedBeads (Cytiva Life Sciences, 45152105050250 and 65152105050250), following the manufacturer's protocol. Eluted peptides were analyzed by LCMS/MS. Samples (~100 ng per injection) were separated on an analytical column (C18 ReproSil AQ, 1.9 µM particle size, 100mm length, 75 µM internal diameter) at 0.5 µL/min with a nanoElute LC system connected in line to a Bruker TimsTOF Pro 2. The column temperature was maintained at 40°C. Peptides were eluted via a water/acetonitrile gradient (buffer A = 0.1% formic acid/water, buffer B = 0.1% formic acid/acetonitrile; flow rate: 0.5 µL/min; gradient: 2% B to 35% B in 0-20 minutes, 35% B to 95% B in 20-20.5 minutes, 95% B in 20.5-22.75 minutes). Scans were performed in positive ion, dia-PASEF mode over a m/z range of 100-1700 with a ramp time of 100 ms, Accu. time of 100 ms, a duty cycle of 100%, a ramp rate of 9.43 Hz, and MS averaging set to 1. Absolute thresholds were set to 10 for MS peaks, and 5000 for mobility peaks.

### 1.2.18 CETSA data analysis

Data was processed via DIANN 1.8.1. Parameters were set as follows: trypsin/P digestion, 3 missed cleavages, 3 max. variable modifications, N-term M excision, Ox(M), Ac(N-term) and C carbamidomethylation. Peptide length range was 7-30, precursor charge range 1-4, m/z range 300-1800, and fragment ion range 200-1800. Mass accuracy and MS accuracy were both set to 10. The following settings on the algorithm were checked: “Use isotopologues”, “MBR”, “No shared spectra”, “Heuristic protein inference”. Precursor FDR was set to 1%. A spectral library was generated via DIANN from all known human proteins (In-Silico spectral library). Resulting matrix.pg file was opened in Perseus (v2.0.7.0). Intensities were imputed as “main”, and the rest of the descriptors were categorical. Data was then transformed (Log base 2). Data was annotated as either “ES5” or “DMSO” sample, grouping all temperature fractions under the same sample annotation. Missing values were imputed with Perseus default settings. Normalization was performed via median subtraction. Following this process, a volcano plot was generated utilizing a t-test for statistical significance. The resulting volcano plots were plotted in GraphPad Prism 10.

### 1.2.19 Membrane remodeling assays

Membrane remodeling of ANXA6 and Syt1 is characterized using isenND[1]. Expression and purification for ANXA6, Syt1 and isenND scaffold proteins were performed as described previously[2,3]. Briefly, BL21 cells expressing these proteins were disrupted and clarified by ultracentrifugation at 50, 000 x g for 45 mins. Cell lysates were then loaded onto an 1ml $Ni^{2+}$-NTA column (Cytiva) for Histag affinity purification. Purified proteins were further fractionated by size exclusion chromatography in reconstitution buffer (50 mM Tris-HCl, pH 8.0; 100 mM NaCl, 5% glycerol, 0.5 mM DTT). For membrane remodeling assays, purified ANXA6 or Syt1 (5 μM) was incubated with isenND (0.5 μM) prepared with PC/PS or PC only lipids in the presence of $CaCl_2$ (0.1 mM) and the indicated concentrations of ES5 in reconstitution buffer. Fluorescence emission of isenND was recorded using a Synergy H1M plate reader (BioTek). Data analysis was performed using GraphPad Prism 10.

## 2. Validation of *in vitro* transcribed mRNA and LNP formulation.

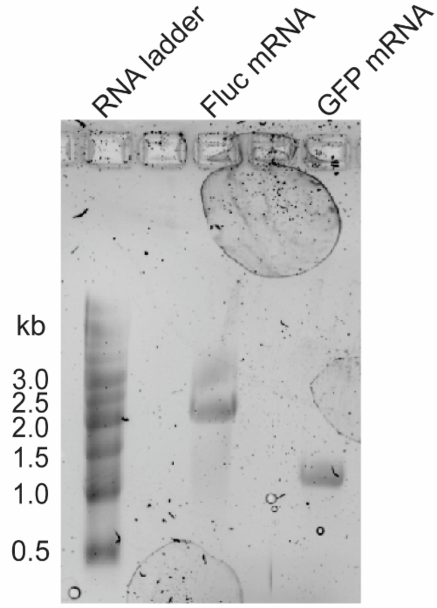


**Figure S1. Characterization of Fluc and GFP mRNAs used in this study.** mRNAs were *in vitro* transcribed and analyzed on a denaturing gel.

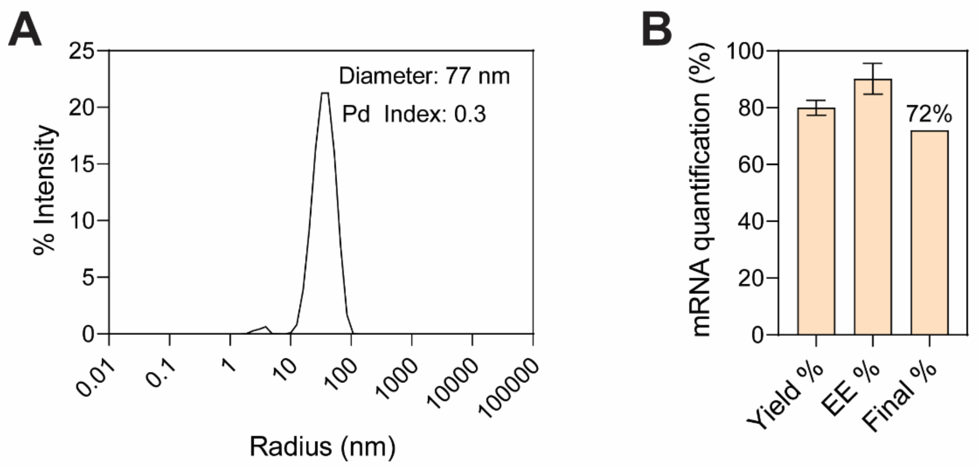


**Figure S2. Properties of mRNA-LNPs used in this study.** (**A**) Histograms of mRNA-LNP showing the average diameter derived from dynamic light scattering measurements. (**B**) Encapsulation efficiencies of mRNA-LNPs quantified using the ribogreen RNA assay. Yield% = $(\text{total mRNA})_{\text{final}}/(\text{total mRNA})_{\text{initial}}$ x100%; encapsulation efficiency (EE%) = $(\text{mRNA})_{\text{encapsulated}}/(\text{total mRNA})_{\text{final}}$ x 100%; Final%= $[(\text{total mRNA})_{\text{final}}/(\text{total mRNA})_{\text{initial}}]$ x $[(\text{mRNA})_{\text{encapsulated}}/(\text{total mRNA})_{\text{final}}]$ x 100%.

## 3. Small molecule treatment on HEK293T cells

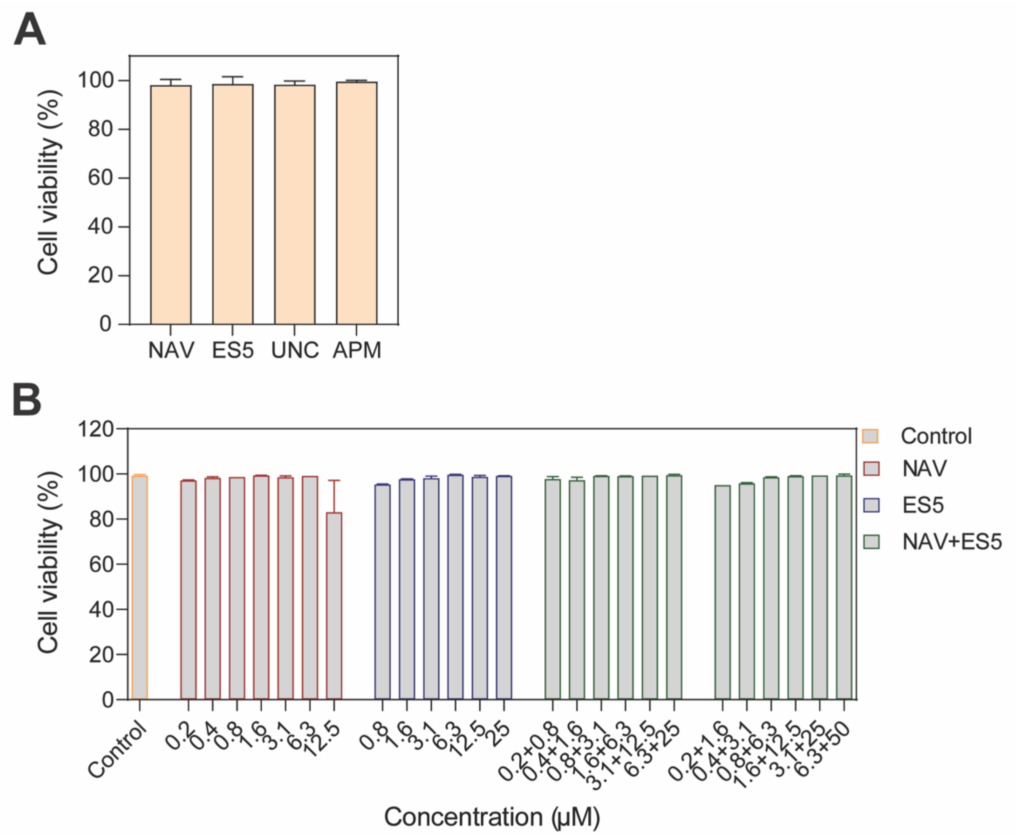


**Figure S3. Impact of small molecules on cell viability.** (**A**) Cell viability determined after the treatment of LNPs harboring Fluc mRNAs and small molecules (NAV, 1.6 μM; ES5, 6.3 μM; UNC, 1.3 μM; APM, 0.01μM). (**B**) Cell viability measurements after the treatment of NAV and ES5 at the indicated concentrations. Mean values are indicated (n=2).

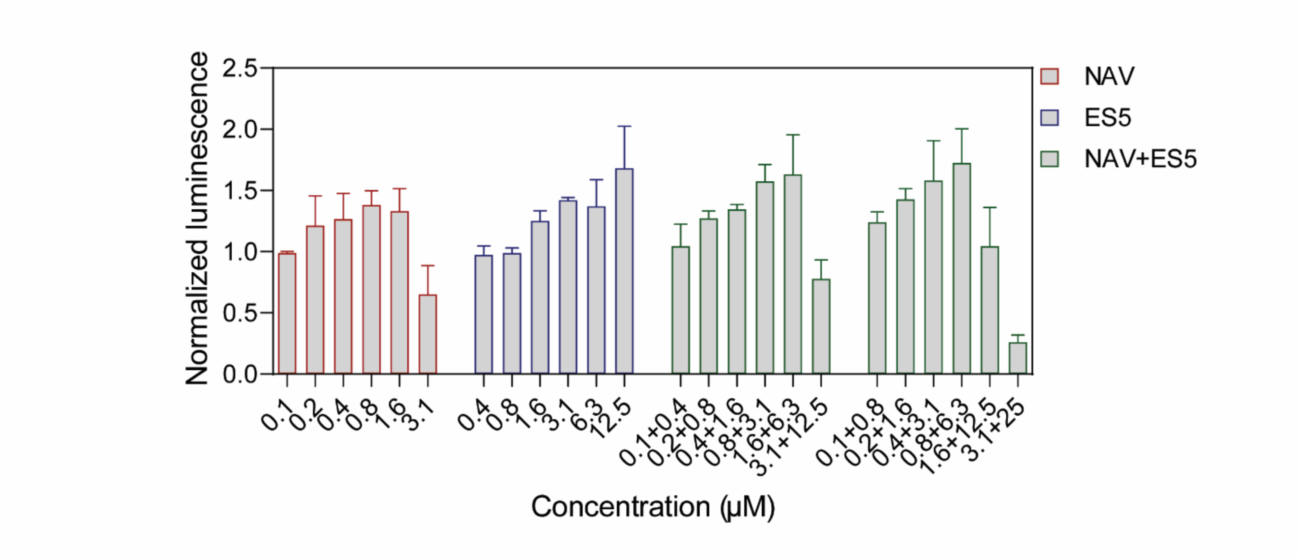


**Figure S4. Impact of small molecules on the delivery of Fluc mRNA-LNPs.**
Expression of Fluc mRNAs delivered by LNPs in HEK293T cells treated with the indicated

amounts of small molecules. Data obtained with small molecules at the indicated concentrations were normalized to control experiments carried out with Fluc mRNA-LNPs alone. Mean values and standard deviations are indicated (n=3).

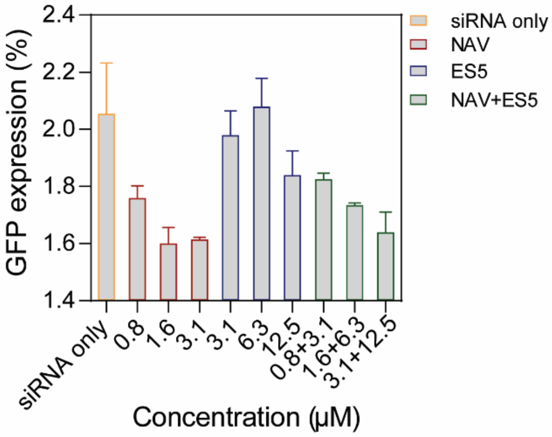


**Figure S5**. **Effects of ES5 and NAV on the efficiency of siRNA-mediated knockdown.** siRNA delivery efficiencies were assayed using a siRNA specific for GFP expressed in a stable HEK293T cell line. Experiments were carried out in cells with or without (siRNA only) treatment of small molecules at the indicated concentrations. Fluorescence signals of the expressed GFP in these cells were quantified by flow cytometry and were normalized to the results obtained from cells that did not receive siRNA in the control experiment. Data were collected from two independent experiments and are shown as mean ± s.d.

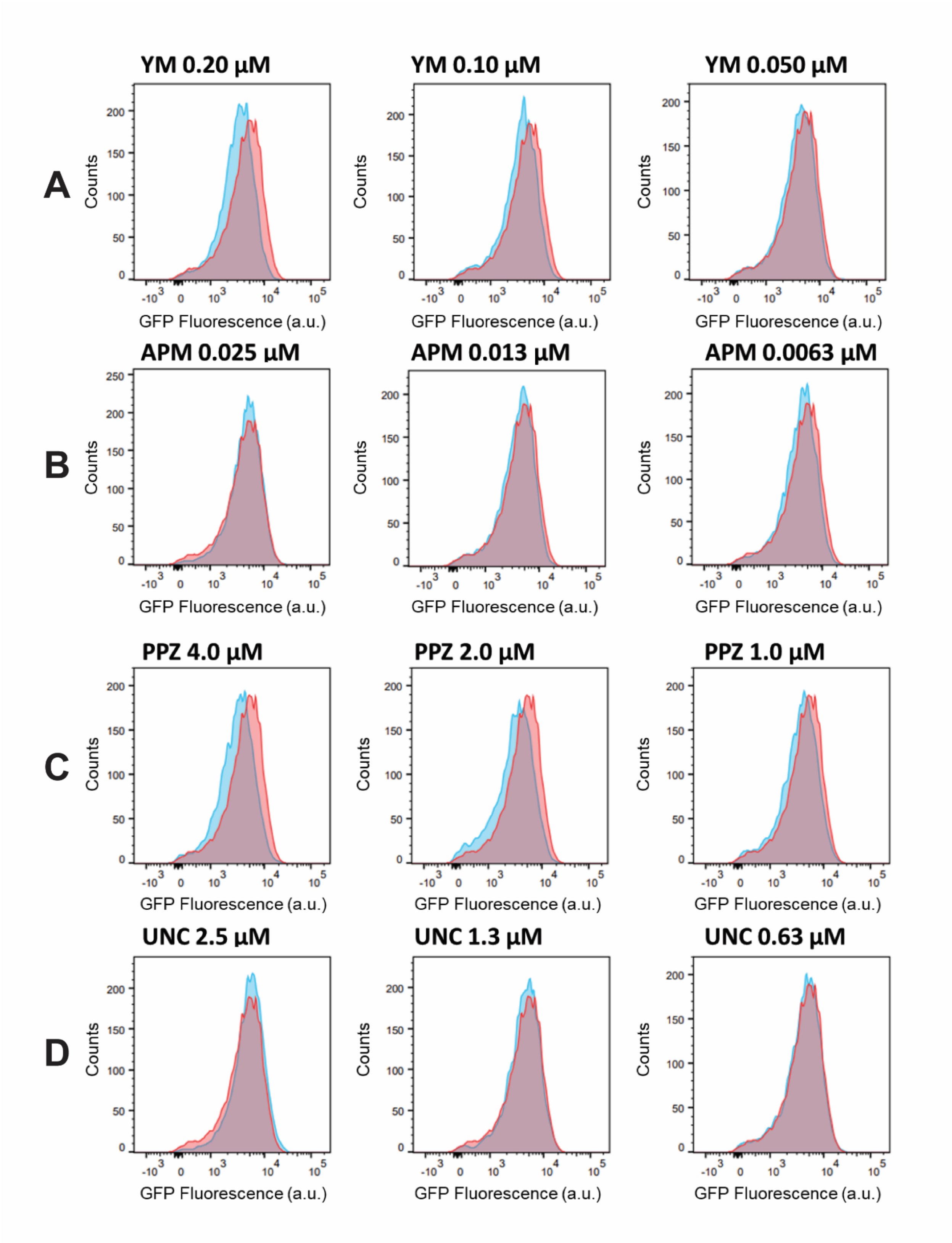


**Figure S6. Small molecules targeting late endosomes are not effective to potentiate mRNA-LNPs.** Histograms of flow cytometry data monitoring the expression of GFP mRNAs delivered by LNPs in HEK293T cells treated with (cyan) or without (red) the indicated amounts of small molecules.

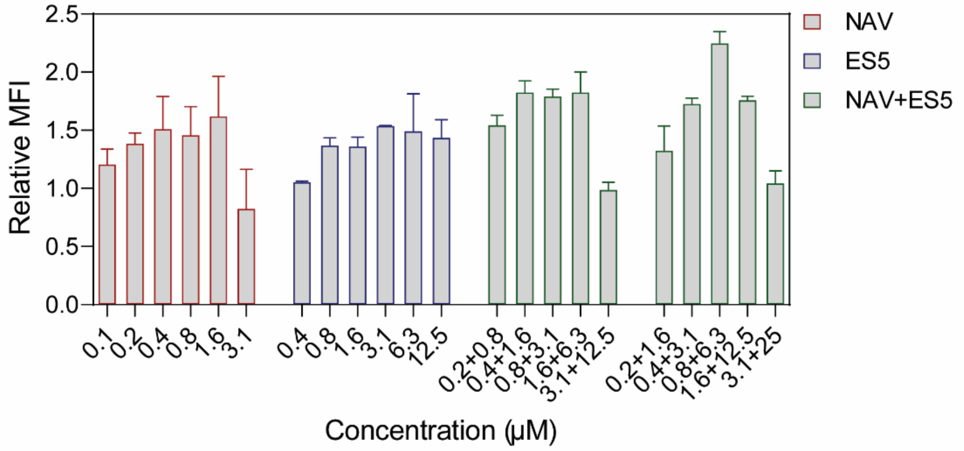


**Figure S7**. **Impact of small molecules on the delivery of GFP mRNA-LNPs.** Expression of GFP mRNAs delivered by LNPs in HEK293T cells was quantified by flow cytometry. Experiments were performed in cells treated with NAV and ES5 at the indicated concentrations alone or in combination. Data obtained from cells treated with small molecules were normalized to control experiments conducted in cells without small molecule treatment. Mean values and standard deviations are indicated (n=3).

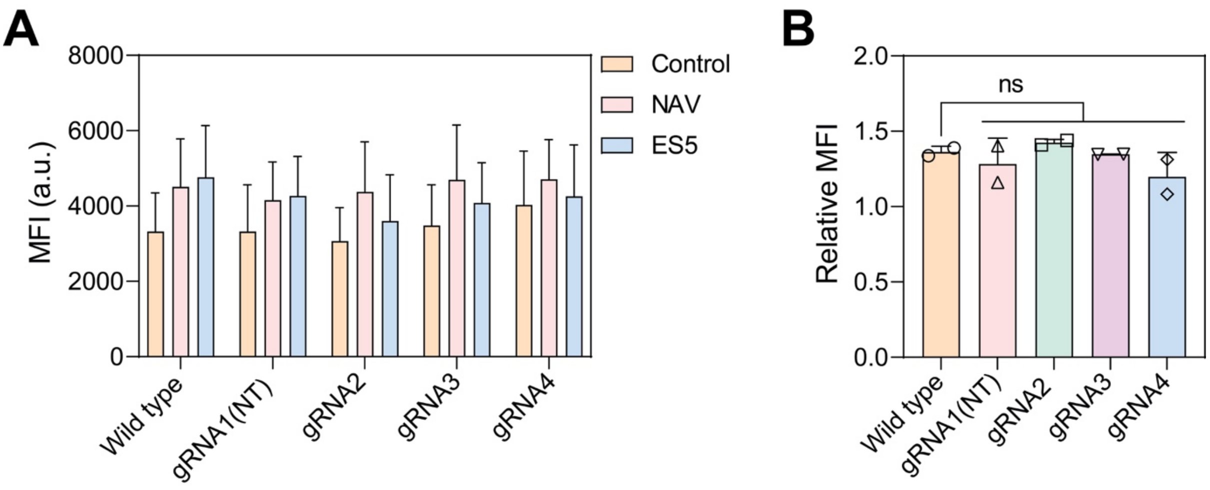


**Figure S8. Impact of NAV and ES5 on mRNA delivery in ANXA6 KO cells.**
(**A**) Quantification of the mean GFP fluorescence signals from wild type and ANXA6 KO cells treated with GFP mRNA-LNPs in the absence and presence of NAV (1.6 μM) and ES5 (6.3 μM). Three specific gRNAs (2-4) were assayed to KO ANXA6 by CRISPR-Cas9 as shown in Figure 4A. In control experiments, we also performed parallel experiments using a non-targeting (NT) gRNA1. (**B**) Data from panel A were further normalized to quantify the stimulatory effect of NAV in wild type and ANXA6 KO cells. The effects of ES5 are shown in Figure 4B. Data are shown as mean ± s.d. Statistics of relative fluorescence intensities per treatment were compared to the control groups using unpaired two-tailed Student's t-test (ns, not significant).

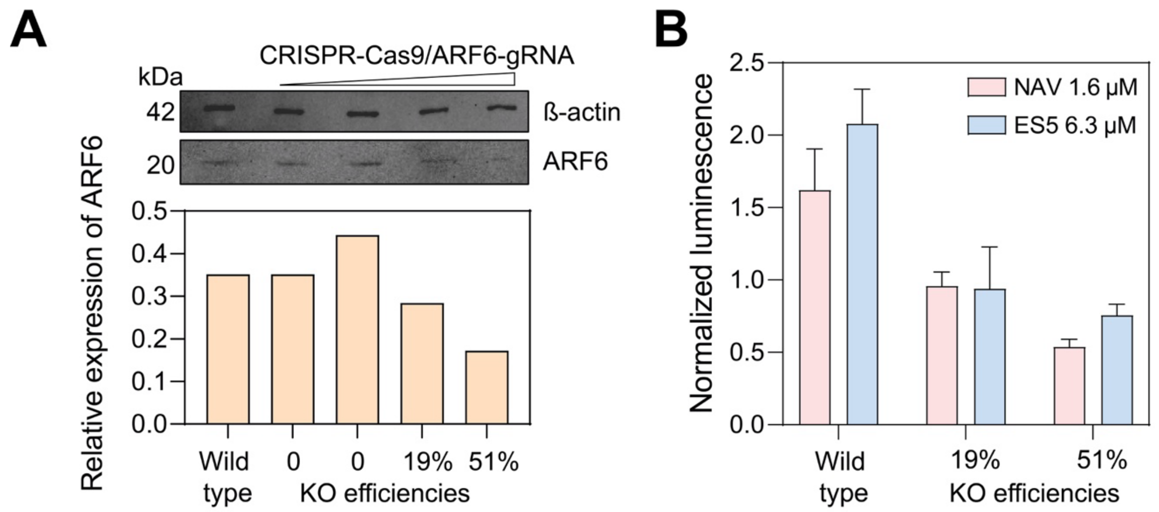


**Figure S9. Effects of ES5 and NAV on the expression of mRNA-LNP in ARF6 KO cells.** (**A**) Analysis of the KO efficiency of ARF6 in HEK293T cells by immunoblot. The expression levels of ARF6 relative to ß-actin were quantified at each condition. (**B**) Characterization of NAV and ES5-stimulated delivery of mRNA-LNPs in ARF6 KO HEK293T cells. Data obtained with small molecules at the indicated concentrations were normalized to control experiments carried out with mRNA-LNPs alone. Mean values and standard deviations are indicated (n=3).

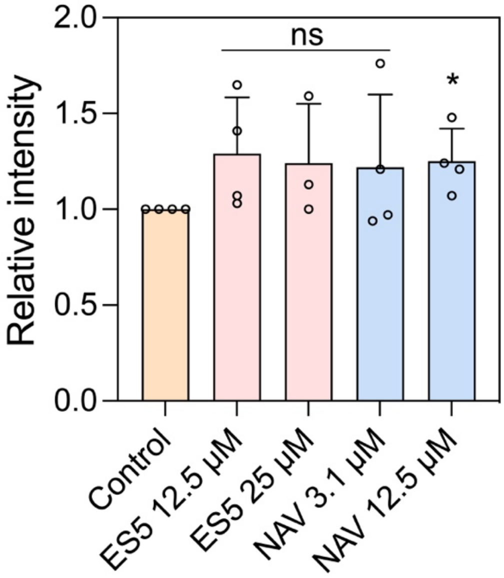


**Figure S10**. **Characterization of NAV and ES5 *in vivo.*** Bioluminescence of Balb/C mice that were intramuscularly (IM) injected with Fluc mRNA-LNPs along with the respective concentrations NAV and ES5. Bioluminescence was quantified and normalized to control experiments without small molecules. Each dot represents an independent experiment (n = 2-3 per group). Statistics of relative bioluminescence intensities per each treatment were analyzed using unpaired two-tailed Student's t-test (* = $p < 0.05$; ns = not significant). Data are shown as mean ± s.d. Results of ES5 and NAV at 12.5 μM are also shown in Figure 5.

## 4. Sequence information

**Table S1.** gRNA sequences utilized in the study

| | |
|---|---|
| gRNA 1 (non-target, NT) | GCCCCGCCGCCCTCCCCTCC |
| gRNA 2 | AGCCTCCAGGTCCCGCTCG |
| gRNA 3 | GACATCATCGGCGACACCTC |
| gRNA 4 | TAATCACCTCACGGAGCAAC |

gRNA 2-4 are specific for Annexin A6, whereas gRNA1 is unspecific and used as a negative control.

## References


1. Ren, Q., Zhang, S. & Bao, H. Circularized fluorescent nanodiscs for probing protein-lipid interactions. *Commun Biol* **5**, 507 (2022).
2. Bao, H. et al. Dynamics and number of trans-SNARE complexes determine nascent fusion pore properties. *Nature* **554**, 260-263 (2018).
3. Zhang, S. et al. One-step construction of circularized nanodiscs using SpyCatcher-SpyTag. *Nat Commun* **12**, 5451 (2021).
